# Apothecaries

**Published:** 1847-09

**Authors:** 


*Apothecaries.*—The following article, which we find in the columns of the *New York Annalist*, refers to a practice too common with apothecaries, which should be rebuked, and, if possible, prevented. The apothecary looks to the physician for his countenance and support, as the only efficient mode of acquiring the confidence of the public, and, in return, not content with the duties of the compounder of medicines, endeavors to appropriate, whenever opportunity permits, the functions of the practitioner. We have a right to complain of this, not chiefly because it diminishes the amount of business which should properly go to the physician, but because, in so far as it has any effect upon the sentiment of the community, it tends to create an impression that the art of prescribing is merely incidental to a knowledge of drugs and pharmacy, instead of the latter being a collateral and subordinate branch of medical science.

As the author of the communication referred to properly remarks, medical men, in their capacity of curators of the interests of the public, are bound to oppose this encroachment, since much injury to health, without doubt, results from it.

We could mention other abuses which have come under our observation, as, doubtless, they have under that of other practitioners. One of these is the habit of exorbitant charging. The public cannot be expected to judge of the propriety of the charges for medicines prescribed. Dependence must necessarily be placed upon the honor or honesty of the apothecary ; and the opportunity for gratifying cupidity sometimes leads him to abuse this trust. If we assume the responsibility of recommending patients to carry their prescriptions to certain establishments, it certainly devolves upon us to endeavor to protect them against imposition. Hence, exorbitant charges by the apothecary should properly come under the cognizance and animadversion of the physician. A direct personal injury is here done to the practitioner, for it will often happen that the pockets of our patients are in that way pretty thoroughly emptied, so that there is little or nothing left for the remuneration of the medical attendant!

Another evil is the habit of making prescriptions subjects of comment or criticism. We have known instances in which the apothecary takes occasion to inquire concerning, or, to show his shrewdness, guess the disease, symptoms, &c., for which the prescription is given, delivering his opinion on its adaptness, &c.

The vending of secret, patented nostrums ought to be disconnected from the legitimate business of the apothecary. We entertain sufficient confidence in the progress of society to believe that the time will come when public sentiment, or the law, or both, will interdict the sale of remedies, the composition of which is concealed. But, before that time arrives, it would seem to be the duty of the medical profession, appreciating, as they do, the nature and extent of the impositions practiced upon the community, to do what they can to discountenance and prevent it. It would do something toward this object, and, at least, be more consistent with dignity and self-respect, to make it a matter of principle to recommend to public favor, and patronize those establishments only which decline to engage in this unholy traffic, provided such establishments are to be found, or can be instituted. While the sale of empirical compounds continues to be sanctioned, let it be confined to shops devoted expressly to the business, and we shall not then, as a profession, be chargeable with indirectly sustaining quackery, by contributing our custom and influence to the support of shops which derive a considerable share of their revenue from that source.

The sale of lottery tickets is interdicted in many of the States of the Union, in order to protect the credulous and infatuated against a foolish expenditure of money, in the hope of a lucky windfall of wealth. With how much more propriety should the law interpose to prevent not only an equally foolish throwing away of money, but the danger of losing what wealth cannot recover, viz., health and life! We have said, that we cannot but think the time will come when public sentiment will require the interposition of effective means to accomplish the latter object. Were we asked to refer to any evidences of an approximation to this period, we confess we should be at a loss to comply. The inordinate desire to be duped seems to be increasing instead of diminishing; and, perhaps, there is encouragement in this fact, on the principle that by satiety of indulgence, the "appetite may sicken and so die;"—but we will not detain the reader longer from the article which has suggested the foregoing remarks:—

"To the Editor of the Annalist:

Sir,—It is a matter of much surprise to me, as I know it is to yourself, that so little interest is manifested by the profession at large in reference to the exposure of quackery, and many kindred evils which operate so injuriously to their interests. One of these kindred evils I wish to bring to your notice, and through your valuable periodical to introduce the same to the particular attention of my professional brethren. I mean the very general practice of our apothecaries in prescribing for, and administering to, individuals who choose to consult them. This is an evil of which all have reason to complain, and it is one which I think the Academy of Medicine would do well to notice, with the view of taking certain steps toward its removal. Go into any apothecary's shop you please, and remain there half an hour or so, and you will almost always find some one come in to consult the apothecary, who never lets him go away without a dose of some sort. This is a common practice among the fraternity, and is one which is fraught with serious evils; for these persons are not always competent to prescribe, if they are to compound. Not unfrequently great mistakes are made by them, in prescribing either the wrong remedy, or in giving an over dose, and death may be the consequence. The apothecary has no just right thus to interfere with what strictly belongs to the physician. His business is to provide himself with the purest drugs, and to compound them according to prescribed formulæ. It is not necessary for him to be thoroughly acquainted with their particular properties, doses, &c., though some knowledge of this kind is useful to him, as well as to the physician who employs him.

I hope that, as the profession has of late manifested a disposition to maintain its dignity, and to advance its interests by a bold and decided opposition to whatever bears the least relation to quackery, it will see the propriety—nay, the necessity of adopting some measures against this encroachment on one of its principal prerogatives.

Let but unanimity of sentiment and unity of action prevail, and in due season the object will be accomplished. MEDICUS."

